# Linezolid-Associated Optic Neuropathy in Drug-Resistant Tuberculosis Patients in Mumbai, India

**DOI:** 10.1371/journal.pone.0162138

**Published:** 2016-09-09

**Authors:** Salil Mehta, Mrinalini Das, Chinmay Laxmeshwar, Sylvie Jonckheere, Sein Sein Thi, Petros Isaakidis

**Affiliations:** 1 Ophthalmology Department, Lilavati Hospital and Research Centre, Mumbai, India; 2 Médecins Sans Frontières, Mumbai, India; 3 Médecins Sans Frontières, Operational Research Unit, Luxembourg city, Luxembourg; Bascom Palmer Eye Institute, UNITED STATES

## Abstract

**Background:**

Patients on linezolid-containing drug-resistant TB (DR-TB) regimen often develop adverse-events, particularly peripheral and optic neuropathy. Programmatic data and experiences of linezolid-associated optic neuropathy from high DR-TB burden settings are lacking. The study aimed to determine the frequency of and risk-factors associated with linezolid-associated optic neuropathy and document the experiences related to treatment/care of DR-TB patients on linezolid-containing regimens.

**Methods:**

This was a retrospective cohort study using routine clinical and laboratory data in Médecins Sans Frontières (MSF) HIV/DR-TB clinic in collaboration with Lilavati Hospital & Research Center, Mumbai, India. All DR-TB patients on linezolid-containing treatment regimens were included in the study and underwent routine evaluations for systemic and/or ocular complaints. Ophthalmological evaluation by a consultant ophthalmologist included visual-acuity screening, slit-lamp examination and dilated fundus examination.

**Results:**

During January 2013-April 2016, 86 of 136 patients (with/without HIV co-infection) initiated linezolid-containing DR-TB treatment. The median age of these 86 patients was 25 (20–35) years and 47% were males. 20 percent of them had HIV co-infection. Of 86, 24 (27.9%) had at least one episode of ocular complaints (the majority blurred-vision) and among them, five (5.8%) had optic neuropathy. Patients received appropriate treatment and improvements were observed. None of the demographic/clinical factors were associated with optic neuropathy in Poissons or multivariate binary logistic-regression models.

**Discussion:**

This is the first report focusing on optic neuropathy in a cohort of complex DR-TB patients, including patients co-infected with HIV, receiving linezolid-containing regimens. In our study, one out of four patients on linezolid had at least one episode of ocular complaints; therefore, systematic monitoring of patients by primary physicians/nurses, and access to specialized diagnostic-services by specialists are needed. As linezolid will be increasingly added to treatment regimens of DR-TB patients, programmes should allocate adequate resources for early diagnosis, prevention and management of this disabling adverse event.

## Introduction

Linezolid, a synthetic oxazolidinone antibiotic, has been shown to be efficacious in the treatment of mycobacterial infections, including multi-drug-resistant tuberculosis (MDR-TB, defined as tuberculosis resistant to rifampicin and isoniazid) [1]. It has shown a significant treatment benefit in two randomized-controlled trials and in small cohorts of MDR-TB patients, with this benefit being most pronounced in patients with additional resistance to fluoroquinolones and injectable anti-TB agents [2, 3]. In the most recent treatment guidelines by the World Health Organization (WHO, 2016) linezolid is recommended as a core second-line drug in the MDR-TB regimen [4].

Patients on linezolid should be under close monitoring for adverse events, particularly anaemia, peripheral and optic neuropathy and lactic acidosis as these can be severe and life threatening. Linezolid inhibits bacterial protein synthesis but it has no major effect on the protein synthesis in mammalian cells. However, intracellular mitochondria are affected by linezolid and long-term administration of the drug may affect protein synthesis. It has been hypothesized that optic and peripheral neuropathy may potentially be the result of this mitochondrial dysfunction [5, 6].

Data on the frequency of linezolid-associated optic neuropathy are still relatively limited and rather inconclusive. Earlier studies of small cohorts and case series have reported relatively low prevalence of optic neuropathy among patients on linezolid ranged between 1.3% and 3.3% [7–9]. However, two recent meta-analyses have reported significantly higher prevalence at 13.2% (10/76 patients) in 2012 and 8% 923/246) in 2015 [10–11]. Programmatic data and experiences from high drug-resistant TB (DR-TB) burden settings are still lacking and the cascade of care of patients on linezolid is still not well described.

Linezolid has recently come off patent and the traditionally high price of this drug is expected to drastically drop due to availability of generic products. It is also expected that the reduced price in combination with the increasing evidence on linezolid’s efficacy and safety and the new WHO treatment guidelines will lead in a dramatic increase in the global use of this drug. It is therefore important to report safety data, as well as programmatic experiences with the ophthalmological monitoring of DR-TB patients on linezolid-containing regimens.

The aim of this study was to determine the frequency of and risk factors associated with linezolid-associated optic neuropathy and document the experiences related to treatment and care of a cohort of DR-TB patients on linezolid-containing regimens in Mumbai.

## Methods

### Ethics

The study has satisfied the criteria for reports using programmatic data, set by the Médecins Sans Frontières independent Ethics Review Board (MSF ERB), Geneva, Switzerland. Since the data used in the study were routinely collected, informed consent of the patient was not obtained. The MSF ERB specifically approved the study and waived the need for consent.

### Study design

This study was a retrospective cohort study using routine clinical and laboratory data.

### Study setting

Médecins Sans Frontières (MSF) in Mumbai, India has been providing treatment and care to HIV and/or DR-TB patients since 2006, described elsewhere [12, 13]. All patients are managed on an out-patient basis by a multi-disciplinary team of trained clinicians, nurses, psychologists, counselors and social workers. Consultant clinical specialists (ophthalmologist, psychiatrist, gastroenterologist etc.) are contacted when needed during treatment and follow-up of the enrolled patients. Patients in need of ophthalmological evaluation were referred to a consultant ophthalmologist at Lilavati Hospital & Research Center, Mumbai, India, a large tertiary health care center.

### Study population

All DR-TB patients, with or without HIV co-infection, who were initiated on linezolid-containing treatment regimens between January 2013 and April 2016 were included in this study.

### Patient follow-up and monitoring

#### Clinical evaluations

Systemic evaluation was conducted as per the clinic follow-up protocol: this consisted of clinical assessment and laboratory monitoring, including complete and differential blood counts, hepatic and renal function tests, HIV markers (subtype of infection, viral load and CD4-counts), sputum collection or extrapulmonary biopsy as necessary. All collected samples underwent molecular studies, culture and drug susceptibility testing for first and second line anti-TB drugs. Appropriate radiography was also carried out. All patients on linezolid-containing regimens underwent a detailed clinical evaluation: patients were asked for systemic and ocular complaints. Visual acuity examination and Ishihara test was carried out by non-specialist clinicians on a routine basis. All self-reported symptomatic patients and all patients with positive findings during the clinical evaluation were referred for ophthalmological evaluation.

#### Ophthalmological evaluations

Patients underwent a full ophthalmological evaluation by a consultant ophthalmologist including visual acuity screening, slit lamp examination and dilated fundus examination of the entire retina in all patients using an indirect ophthalmoscope.

Optical coherence tomography was done whenever deemed necessary by the physician.

### Data collection and analysis

Data were routinely collected during each consultation and entered into handwritten patient files and an electronic database.

Self-reported symptomatic patients and patients with positive findings during the clinical evaluation were defined as “presumptive linezolid-associated optic neuropathy”, while patients with “linezolid-associated optic neuropathy” were diagnosed by a specialist ophthalmologist.

Patient characteristics were summarized using descriptive statistics. We used t-test, chi-square or Fisher’s exact test to assess differences of variables between groups, as appropriate. To identify factors associated with linezolid-associated neuropathy in DR-TB patients, bivariate and multivariate analyses were performed using Poisson and binary logistic regression models. A p-value of less than 0.05 was considered to indicate statistical significance. SPSS (Version 20.0, Armonk, NY: IBM Corp. Released 2011) was used for analysis.

## Results

### Patient characteristics

Between January 2013 and April 2016, 136 patients (with or without HIV co-infection) initiated DR-TB treatment. Of these, 86 had linezolid in their treatment regimen. The median age of these patients was 25 (20–35) years and 47% were males. 20 per cent of the patients were co-infected with HIV and 42% had DR-TB that was also resistant to a fluoroquinolone or an aminoglycoside.

**Fig 1:**
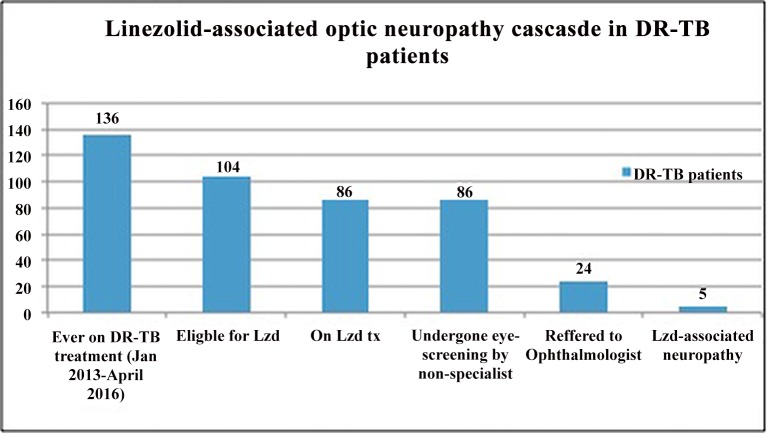
Linezolid-associated optic neuropathy cascade in DR-TB patients, Mumbai, India.

Of these 86 DR-TB patients on linezolid, 24 (27.9%) had at least one episode of ocular complaints (the majority blurred vision) and were referred to the consultant ophthalmologist. Of these, five patients (5.8%, 95% Confidence Intervals: 0.9% to 10.7%) were diagnosed with optic neuropathy. The diagnosis cascade of patients on linezolid-containing regimens in this Mumbai cohort is shown in Fig 1.

Clinical characteristics of patients on linezolid-containing regimens are presented in Table 1 while characteristics of patients with linezolid-associated optic neuropathy are shown in Table 2. None of the factors were associated with ‘presumptive linezolid-associated optic neuropathy’ or ‘linezolid-associated optic neuropathy’ in Poissons or multivariate binary logistic regression models.

**Table 1:** Demographic and clinical characteristics of drug-resistant tuberculosis patients on Linezolid-containing regimen in Mumbai, India, January 2013-April 2016.

Explanatory Variable	DR-TB patients on Lzd-containing regimen (N = 86), n(%)	Patients with presumptive optic neuropathy^┼^ (N = 24), n(%)	Patients without presumptive optic neuropathy^┼^(N = 62), n (%)	Chi-square/ t-test (p-value)
**Age** [years, mean (SD)]	27.5 (9.9)	29.1 (10.1)	26.9 (9.9)	0.94 (0.35)
**Sex of patients**				
Male	40 (46.5)	13 (32.5)	27 (67.5)	0.78 (0.38)
Female	46 (53.5)	11 (23.9)	35 (76.1)	
**HIV co-infection**	17 (19.8)	6 (35.3)	11 (64.7)	0.58 (0.45)
**TB site**				
Pulmonary	80 (93.0)	22 (27.5)	58 (72.5)	0.2 (1.0)
Extra-pulmonary	6 (7.0)	2 (33.3)	4 (66.7)	
**DR-TB resistance pattern**				
PDR or MDR	7 (8.1)	3 (42.9)	4 (57.1)	6.1 (<0.05)
Pre-XDR	36 (41.9)	5 (13.9)	31 (86.1)	
XDR and above	43 (50.0)	16 (37.2)	27 (62.8)	

^┼^Row percentage in parenthesis; Lzd: linezolid; SD: Standard deviation; PDR: Poly-drug resistant; MDR: Multi-drug resistant; Pre-XDR: Pre-extensively-drug resistant; XDR: Extensively drug resistant

**Table 2:** Demographic and clinical characteristics of drug-resistant tuberculosis patients with linezolid-associated optic neuropathy, Mumbai, January 2013-April 2016.

Case	Sex /Age	TB site	DR-TB pattern	Clinical symptoms/ signs	Ophthalmological Evaluation (in either eye)	Treatment regimen	LZD(m)
**1**	F/22	PTB	XDR	- B/L blurring of vision, -S/S of Peripheral neuropathy	-VA: 6/60, NV<N36, -OM & ASE with slit lamp: N, DLE: B/L hyperemic, mildly elevated optic nerve heads, -R&V: N	Capreomycin inj 750 mg OD, levofloxacin 750 mg OD, cycloserine 500 mg OD, Para-amino-salicylate 9gm/day, amoxicilline-clavulanate 625mg TDS, clofazimine 100mg OD, linezolid 600 mg OD), bedaquilline	11
**2**	F/22	PTB	XDR	- B/L blurring of vision-S/S of Peripheral neuropathy	-VA: 6/24, NV<N36, -OM & ASE with slit lamp: N, -DLE: B/L mildly hyperemic, elevated optic nerve heads-R&V: N	Capreomycin 1g OD, Ethambutol 800 mg/day levofloxacin 1000 mg OD, cycloserine 500 mg OD, Para-amino-salicylate PAS, 9.2 gm/day, clofazimine 100 mg OD, amoxycilin-clavulanic acid 625mg TDS, linezolid 600 mg OD	11
**3**	M/25	PTB	MDR	- B/L blurring of vision	-VA: 6/60, NV<N36-OM & ASE with slit lamp: N-DLE: B/L mildly hyperemic, elevated optic nerve heads-R&V: N	levofloxacin 1000 mg OD, cycloserine 500 mg OD, Para-amino-salicylate 9.2 gm/day, clofazimine 100 mg OD, amoxycilin-clavulanic acid 1000 mg/250 mg/ day, linezolid 600 mg OD	10
**4**	F/29	PTB	XXDR	- B/L blurring of vision	-VA: 6/45 (R), 6/30 (L), NV<N36-OM & ASE with slit lamp: N-DLE: B/L mildly hyperemic, elevated optic nerve heads-R&V: N	Capreomycin inj 750 mg OD, clofazimine 100 mg OD, amoxycilin-clavulanic acid 1000 mg/250 mg/ day, linezolid 600 mg OD	7
**5**	M/32	PTB	XXDR	- B/L blurring of vision	-VA: 6/60, NV<N36-OM & ASE with slit lamp: N-DLE: B/L mildly hyperemic, elevated optic nerve heads-R&V: N	Capreomycin inj 1000 mg OD, ethionamide 750 mg OD, clofazimine 100 mg OD, linezolid 600 mg OD, Delamanid 100mg BD, Imipenem 1g BD, Amoxi-Clav 625mg TDS	11

F: Female; M: Male; PTB: Pulmonary TB; DR-TB: Drug resistant TB; XDR: Extensively drug-resistant; MDR: Multi drug-resistant; XXDR: Extremely drug-resistant; B/L: Bilateral; S/S: Symptoms & Signs; VA: Visual acuity; R: right; L: left; NV: near vision; OM & ASE: ocular motility and anterior segment evaluation; DLE: Dilated fundus examination; R &V: retina & vasculature; OD: once daily; Lzd (m): Duration of Linezolid administration in months

### Ophthalmological findings and patient management

#### Case 1

A 23 year old female presented with bilateral blurring of vision for 15 days. Significant medical history included pain and difficulty in walking, suggestive of a peripheral neuropathy since a few weeks prior to the visual symptoms.

On examination, her best corrected visual acuity was 6/60 and near vision < N36 in either eye. Examination of ocular motility and anterior segment evaluation with a slit lamp were normal. Dilated fundus examination revealed hyperemic, mildly elevated optic nerve heads bilaterally. The retina and vasculature were normal. A preliminary diagnosis of optic neuropathy, presumably due to linezolid toxicity was suggested. She underwent fundus photography and an optical coherence tomography (OCT, RNFL program, Stratus OCT, Carl Zeiss Meditec Inc., Dublin, CA) of the optic nerve head bilaterally but declined to undergo perimetry.

She was started empirically on oral prednisolone (40 mg daily tapering by 10 mg weekly), linezolid was discontinued and the patient was followed up. She underwent repeat OCT on day 8 and on day 22. By her last follow up her visual acuity improved to 6/6 bilaterally with N6 near vision. There was a marked reduction of the optic nerve head swelling to near normalcy.

At the first OCT examination (RNFL), there was a generalised increase in the RNFL thickness in all quadrants, which rapidly decreased over the follow up period.

After 10 weeks of interruption, Linezolid 300mg OD was re-introduced and patient was doing well during the treatment.

#### Case 2

A 24-year-old female patient presented with bilateral blurring of vision for 10 days and paresthesia and pain in both hands (suggestive of a peripheral neuropathy).

On examination, her best corrected visual acuity was 6/24 and near vision < N36 in either eye. Examination of ocular motility and anterior segment evaluation with a slit lamp were normal. Dilated fundus examination revealed mildly hyperemic and elevated optic nerve heads bilaterally. The retina and vasculature were normal. A preliminary diagnosis of linezolid induced optic neuropathy with an additional differential diagnosis of ethambutol toxicity. She underwent a perimetry (SITA fast, central 30–2, Carl Zeiss Meditec Inc., Dublin, CA), fundus photography and an optical coherence tomography (OCT) of the optic nerve head bilaterally.

She was started empirically on oral prednisolone (40 mg daily tapering by 10 mg weekly) and was followed up. Ethambutol was discontinued permanently, and linezolid was interrupted for about four months. She underwent repeat OCT on day 18 and on day 25 and repeat perimetry on day 45. During the last follow-up (day 45) of the patient, visual acuity has improved to 6/6 bilaterally with N6 near vision. There was a marked reduction of the optic nerve head swelling to normalcy.

**Fig 2:**
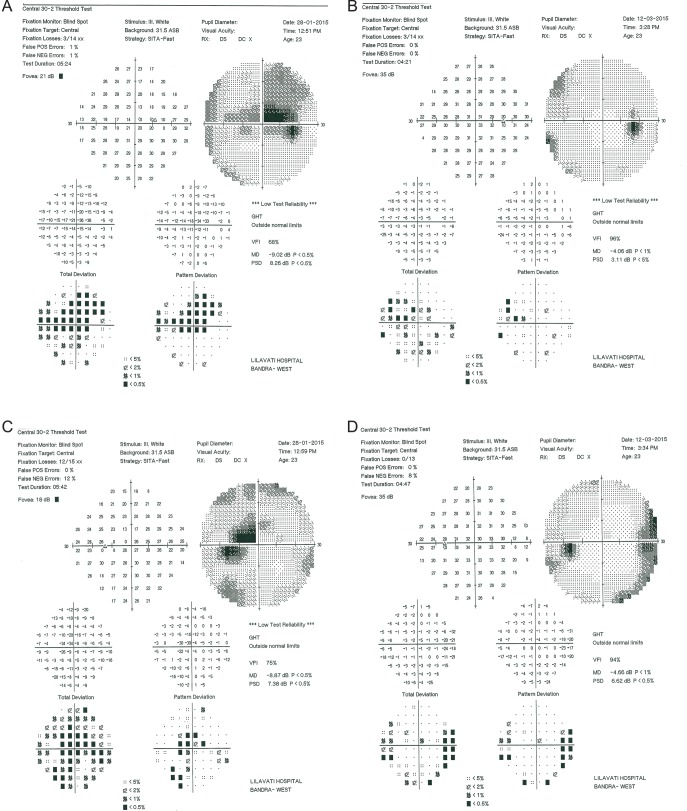
Perimetric Findings of Case 2; A) Right eye at diagnosis showing centrocecal scotomas with an associated bitemporal central scotoma and B) Right eye at day 45 showing resolution. Similar findings are seen in the Left eye at diagnosis (C) and at day 45 (D).

The OCT test results showed similar findings of generalized RNFL thickness, which rapidly subsided. The visual field analysis performed on the Humphrey 24–2 SITA fast program showed dense central bitemporal defects that respected the vertical midline and had associated cecocentral scotomas bilaterally. There was a rapid reduction in scotoma size and intensity over the follow-up. No recurrence of adverse events after re-initiation of Linezolid 60 mg in the treatment regimen of the patient. The perimetric findings are shown in Fig 2.

#### Case 3

A 25 year old male presented with bilateral blurring of vision for one week and no signs or symptoms of peripheral neuropathy.

The vision as tested on his initial visit was 6/6 bilaterally with a normal appearing fundus. This visual loss rapidly progressed over a week to 6/60 with near vision < N36 in either eye. He was fully reevaluated. Examination of ocular motility and anterior segment evaluation with a slit lamp were normal. Dilated fundus examination revealed mildly hyperemic and elevated optic nerve heads bilaterally. The retina and vasculature were normal.

A preliminary diagnosis of linezolid induced optic neuropathy was made and he was started on oral prednisolone (40 mg daily tapering by 10 mg weekly) and was followed up. Linezolid tablets were discontinued. He underwent a magnetic resonance imaging (MRI) of the brain and orbits with gadolinium contrast and it was normal.

He underwent a perimetry (central 30–2), fundus photography and an optical coherence tomography (OCT) of the optic nerve head bilaterally. He underwent repeat OCT on day 10 and day 45 and a repeat perimetry on day 45.

The vision improved significantly over the next few weeks and at day 45 follow-up he was reading 6/6 with normal N6 visual acuity.

The OCT test results showed similar findings of generalized RNFL thickness, which rapidly subsided. The initial perimetry showed generalized reduction of sensitivity bilaterally along with centrocecal scotomas in the left eye with a central temporal scotoma. No such scotoma was seen in the right eye. There was a rapid reduction in scotoma size and intensity over the follow-up period.

#### Case 4

A 29-year-old female patient presented with a 7-day history of bilateral blurred vision. There were no symptoms or signs suggestive of peripheral neuropathy. She was fully evaluated and examination of ocular motility and anterior segment evaluation with a slit lamp were normal. Her visual acuity was 6/45 in the right eye and 6/30 in the left with <N36 near acuity. Dilated fundus examination revealed mildly hyperemic and elevated optic nerve heads bilaterally. The retina and vasculature were normal. A preliminary diagnosis of linezolid induced optic neuropathy was made and linezolid tablets were discontinued.

She underwent an initial perimetry (central 30–2) and fundus photography. The initial perimetry showed generalized reduction of sensitivity bilaterally along with centrocecal scotomas in either eye along with a central bitemporal scotoma. She underwent a magnetic resonance imaging (MRI) of the brain and orbits with gadolinium contrast but it was normal in all respects.

At day 38 follow-up, her visual acuity had improved to 6/15 bilaterally and N8 for near but there was a worsening of her visual fields bilaterally. A quadrantanopia had developed in the right eye.

On her last follow-up at day 78, her visual acuity was 6/6 bilaterally and a repeat perimetry showed a marked improvement of her left eye to near normalcy and a significant reduction of the scotomas in her right eye.

**Fig 3:**
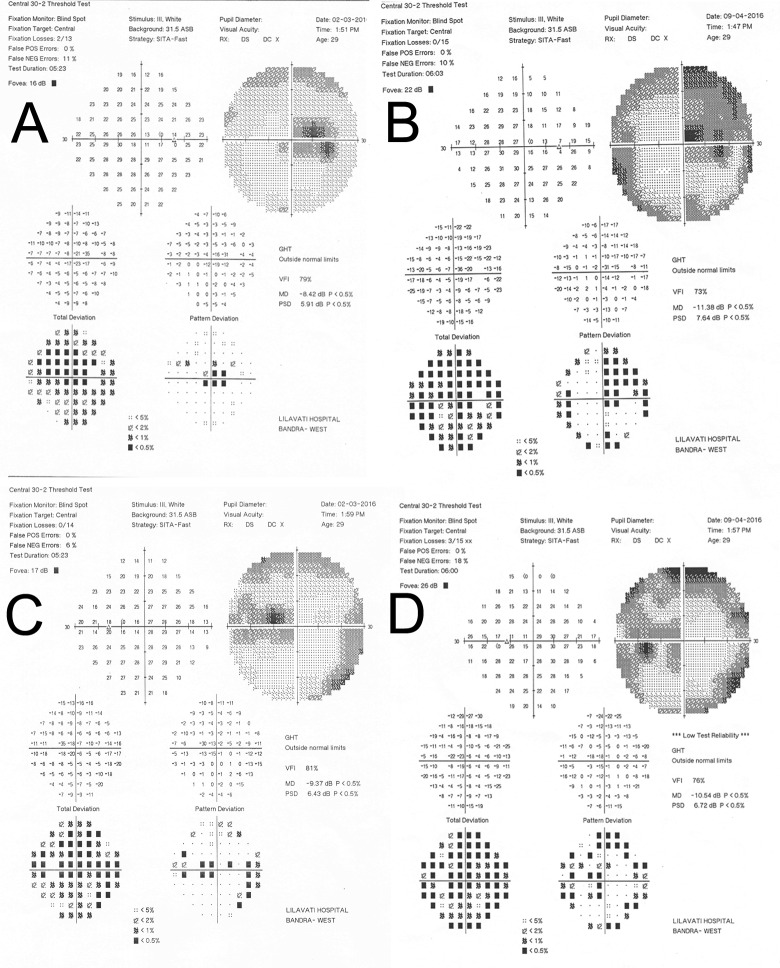
Perimetric Findings of Case 4; A) Right eye at diagnosis showing centrocecal scotomas with an associated bitemporal central scotoma and B) Right eye at day 38 showing worsening. Similar findings are seen in the Left eye at diagnosis (C) and at day 38 (D).

The perimetric findings are shown in Fig 3.

#### Case 5

A 25-year-old male presented with complaints of bilateral blurring of vision for two weeks. The patient reported no symptoms or signs suggestive of peripheral neuropathy.

The vision as tested on his initial visit was 6/60 with near vision < N36 in either eye. Examination of ocular motility and anterior segment evaluation with a slit lamp were normal. Dilated fundus examination revealed mildly hyperemic and elevated optic nerve heads bilaterally. The retina and vasculature were normal. Linezolid tablets were discontinued. There were no symptoms or signs suggestive of peripheral neuropathy.

He underwent an initial perimetry (central 30–2) and fundus photography. He declined to undergo an optical coherence tomography (OCT) of the optic nerve heads. The perimetry showed generalized reduction of sensitivity bilaterally along with centrocecal scotoma in the left eye associated with a central temporal scotoma. No scotoma was seen in the right eye. Linezolid (600mg on alternate days) was re-introduced after 3 weeks of discontinuation. He underwent a magnetic resonance imaging (MRI) of the brain and orbits with gadolinium contrast and it was normal in all respects.

## Discussion

In this study we report relative low rates of linezolid-associated optic neuropathy in a cohort of complex DR-TB patients, including patients co-infected with HIV, receiving linezolid-containing regimens in Mumbai, India. This is among the first studies that report on the programmatic use of linezolid in India and to our knowledge, this is the first detailed report focusing specifically on optic neuropathy among DR-TB patients receiving linezolid.

We describe a satisfactory cascade of treatment and care among patients receiving linezolid in this Mumbai cohort. The clinical team has been systematically monitoring the patients using verbal screening, visual acuity and Ishihara tests; virtually all patients on linezolid-containing regimens have been screened at least once. Having access to specialized ophthalmological services and a consultant ophthalmologist was an essential component in the cascade.

Overall, one out of four patients on linezolid had at least one episode of ocular complaints, usually blurred vision. Of symptomatic patients one in five were finally diagnosed with linezolid-associated optic neuropathy and the overall prevalence of optic neuropathy was 6 per cent. We needed to screen 5 symptomatic patients to find a new case of optic neuropathy (NNS = 5).

Our findings compare with the latest systematic review and meta-analysis of a total of 367 patients on linezolid-containing regimens. Among 246 patients with data on optic neuritis 23 (8%) had optic neuritis and there was no statistically significant difference between high and low dose of the drug, using a cut-off of 600mg/day [11]. A previous meta-analysis of 121 patients has reported higher frequency of optic neuropathy (10/76, 13.2%) [10]. Interestingly, previous case-series and small programmatic cohorts have reported lower prevalence of optic neuropathy among patients on linezolid. Schecter at al reported 1/30 (3.3%) patients with optic neuropathy in a North American cohort, Udwadia and colleagues from Mumbai reported 1/78 (1.3%), while in a previous analysis of the Mumbai cohort pooled with a South African MSF cohort we found 1/34 (2.9%) patients with optic neuropathy [7–9]. The global cohort of patients with DR-TB on linezolid-containing regimens remains very small and we need more data from randomized studies and large programmatic cohorts in order to understand the true magnitude of linezolid toxicity.

In this cohort linezolid neuropathy was presented as an acute, profound and bilateral visual loss but with a varied fundus picture. The visual acuities of the patients ranged from 6/24 to 6/60 while the near vision was <N36 in these five patients. Examination of ocular motility and anterior segment evaluation with a slit lamp were normal. A dilated fundus examination revealed bilateral hyperemic, mildly elevated optic nerve heads in these five patients while the retina and vasculature were normal.

The optical coherence tomography (OCT) showed generalised increases that rapidly improved over the follow up period. This is a common finding in all toxic neuropathies and did not appear to be specific for linezolid toxicity. This increase in the RNFL thickness is due to axoplasmic stasis for the axons of the ganglion cells.

We used the Guided Progression Analysis (GPA) software (ver. 6.0) to study potential RNFL loss over subsequent visits. All three patients demonstrated areas of possible or likely loss. The normative database is relatively small and has been extensively used for open angle glaucoma. No data exists on its applicability in toxic or drug neuropathies and thus, we are unable to comment on whether this constitutes true subclinical RNFL loss as most eyes recovered to normalcy without residual scotomas or is a statistical fallacy. Recently, there has been a resurgence of interest in using GPA in demyelinating optic nerve disease, associated with multiple sclerosis, specifically for prognosticating outcomes. Further data might suggest a role for serial OCTs in drug induced optic neuropathies.

There were consistent perimetric findings and these may reflect toxicity in a specific topographic pattern especially to the papillomacular bundle as well as to chiasmal fibers. Improvement was seen in all patients over a few weeks (usually a month). This was seen in patients who received oral steroids (tapered over a month) as well as those not receiving steroid treatment. Saijo et al. have suggested that oral corticosteroid treatment might be deleterious in these patients but we noted a marked improvement in all treated patients [14]. However, a larger dataset would be needed to allow any definite conclusions on the benefit of steroids in this condition.

We looked at the available literature for data or images to assess the visual field pathologies. Eight studies have described the perimetric findings of 11 patients and this included one patient with unspecified bilateral visual field defects, four patients with bilateral centrocecal scotoma, two with bilateral central scotomas and one patient with quadrantanopia [14–23]. We analyzed the perimetric data of eight eyes of four patients and six eyes demonstrated a distinct pattern of a cecocentral scotoma associated with a central bitemporal scotoma that respects the vertical midline. This is unusual in that most toxic and nutritional optic neuropathies conventionally described lack this finding. None of the authors of studies of linezolid neuropathy have described this distinctive finding so far. There was a rapid decrease in the depth and size of the scotoma over a few weeks with most eyes returning to the baseline.

Similar findings were seen in the visual fields of patients with ethambutol toxicity. Kho et al described the perimetric findings in 19 patients [24]. The majority showed scotomas that were denser in the temporal fields, usually with margination along the vertical midline with associated central or cecocentral scotomas. These were similar to our findings. They postulated that ethambutol might selectively affect the fibers in the chiasm that cross over and recommended neuroimaging but their imaging was normal.

Ethambutol and linezolid appear to have similar and specific perimetric patterns that may reflect a related mechanism of toxicity involving mitochondrial dysfunction.

There are some limitations to this study. First, the study design was retrospective and the data were routinely collected in a specific programmatic setting. Our findings may not be applicable to other populations. Second, the cohort size was small and the finding should be interpreted with caution. However given the small size of the global cohort of DR-TB patients on linezolid-containing regimens we believe that our study contributed a significant amount of data to the evidence-base.

As linezolid will be increasingly added to treatment regimens of DR-TB patients around the world, regular ophthalmological screening may help in early identification of patients with linezolid-associated neuropathy. Clinicians, ophthalmologists, public health specialists and programme managers will increasingly encounter linezolid-associated toxicity, including a small but important proportion of patients with optic neuropathy. Systematic monitoring of patients by primary physicians and nurses, as well as access to specialized diagnostic services by specialists will be needed. Programmes should be prepared and allocate adequate resources in order to early diagnose, prevent and aggressively manage this disabling adverse event.
